# Perceived social support according to the type and number of addictions

**DOI:** 10.1371/journal.pone.0329256

**Published:** 2025-08-11

**Authors:** Alexander Tomei, Joseph Studer, Gerhard Gmel

**Affiliations:** 1 Addiction Medicine, Department of Psychiatry, Lausanne University Hospital and University of Lausanne, Switzerland; 2 Service of Adult Psychiatry and Psychotherapy West, Department of Psychiatry, Lausanne University Hospital and University of Lausanne, Prilly, Switzerland; 3 Centre for Addiction and Mental Health, Institute for Mental Health Policy Research, Toronto, Ontario, Canada; 4 Department of Health and Social Sciences, University of the West of England, Bristol, United Kingdom; Jawaharlal Institute of Postgraduate Medical Education and Research, INDIA

## Abstract

**Background:**

Addiction is very closely related to social determinants. It can be an outcome of the excessive consumption pushed by society and be protected against by society’s many positive dimensions. The main objectives of the present paper are to examine whether perceived social support (PSS) – the perceived availability of others to provide us assistance when needed – varies (a) according to different types of addiction (i.e., gambling, gaming, alcohol, cannabis, and nicotine), and (b) according to the presence of multiple addictions.

**Methods:**

A sample of 5654 male emerging adults (mean age = 21.34 years old) completed a questionnaire that included a scale for measuring PSS and several screening tools used to identify addictive behaviors like gambling, gaming, and the consumption of alcohol, cannabis, or nicotine.

**Results:**

Compared to a non-addicted control group and to peers with alcohol, cannabis or nicotine disorder, male emerging adults with gambling or gaming addiction reported the lowest levels of PSS. Moreover, these findings showed that PSS decreased among male emerging adults with multiple addictions.

**Conclusion:**

Our results suggest that young men with gambling and gaming disorders may be at a greater risk of social isolation and loneliness and, thus, of experiencing deteriorations in their physical and mental health.

## Introduction

Addiction is a pervasive, long-lasting biopsychosocial disorder and a growing public health issue. From 2011–21, worldwide drug use increased by 23%, whereas drug-use-related deaths rose by 17.5% from 2009–19 [1]. Behavioral addictions form a much more recent domain of research such that prevalence comparisons over time remain limited. However, generalized addiction to the internet has increased significantly since the early 2000s [2] as the number of individuals using it keeps growing [3]. The physical and mental consequences of the user’s addiction have long been recognized as a challenge. In the past 15 years, efforts have grown to better understand how addiction is related to social behaviors. Namely, the extent to which individuals with addictive disorders connect to others and maintain adjusted relationships with them.

It is well recognized that a lack of social relationships constitutes a major risk factor for health [4], one as great as other well-recognized risk factors like smoking, blood pressure, lack of exercise, and obesity [5]. Loneliness, for example, has been shown to be a risk factor for cardiovascular disease [6] and for mortality [7]. Indeed, regardless of respondents’ age or socioeconomic status, the likelihood ratio of death increases by 26% for people who report feeling lonely, by 29% for those who are socially isolated, and by 32% for those living alone [8,9].

Numerous studies have investigated the social abilities of people with addiction. Some investigated their ability to empathize—their ability to share others’ emotions and adopt their perspectives. These studies consistently showed reduced levels of empathy both in substance-related addictions [10–16] and in behavioral addictions [17–19]. Other studies have examined how much individuals with addictive disorders exhibit prosocial behaviors, revealing that they engaged in them less than their healthy peers [20,21]. For example, Tomei et al. [21] showed that young males with gambling and gaming disorders reported lower levels of prosocial behaviors than controls with no addiction and their peers with alcohol, cannabis, or nicotine disorder. Furthermore, respondents with a gambling disorder appeared to have the lowest level of prosocialness among the young men with the types of addictions considered.

These findings seem to corroborate earlier ones describing how individuals who had lived through distressing situations tended to alienate others and failed to reciprocate the support they received from them, hence diminishing the providers’ willingness to support them further [22,23]. Indeed, according to social exchange theory, people exchange benefits based on reciprocity [24] and equity [22]: when they provide a benefit to someone, they expect a similar benefit in return. Consequently, any disruption, interruption, or inequity in this exchange of benefits can trigger negative feelings and dissatisfaction about the relationship, potentially leading to providers reducing their social support to recipients.

A reduction or loss of social support may constitute a serious challenge to the recipient’s health. Indeed, whether it is real or perceived, there is now extensive evidence of social support’s impact on both physical and mental health. For example, social support has been shown to reduce physiological stress responses [9] and to alleviate inflammation processes related to diseases such as cancer, cardiovascular diseases, and diabetes [for a review, see 25]. Conversely, low levels of perceived social support (PSS) are associated with an accelerated deterioration of CD4 count in men with HIV [26] and with the development of and a worsening prognosis for coronary heart disease [27]. Regarding mental health, perceived social support is associated with lower levels of depression and anxiety, reduced loneliness [28,29], higher self-esteem, and better sleep quality [30–33]. Reciprocally, poor social support for people with depression has worsened outcomes in terms of symptoms, recovery, and social functioning [34].

The present study investigated how individuals with addictive disorders perceived the social support they received from significant others and friends. More specifically, we examined how PSS varied according to the type of addiction (i.e., substance-related or behavioral) and to having multiple addictions.

Several studies have demonstrated how social support contributes to hindering health-damaging behaviors, such as the use of psychoactive substances [35,36]. For example, greater social support from family members and friends has been shown to induce reductions in the use of alcohol, tobacco, cannabis, and cocaine [37–45]. Moreover, greater PSS was found to be associated with fewer problematic behaviors related to social media use [46], internet use [47,48], gambling [49–52], and gaming [53].

Given their salutary effects on reducing substance use and problematic behaviors, social connections and social support could be used to boost intervention and treatment programs. Evidence on this shows that family and peer support can play a key role in the success of the treatment and recovery of individuals with an opiate addiction [54]. Moreover, treatments that involve family members in the therapeutic process contribute significantly more to reducing substance use than treatments that do not include them [55].

Thus, drawing attention to the associations between social support and addiction is highly relevant. However, understanding those associations better may raise questions about whether social support has the same effects on every type of addiction. Two specific questions appear. Firstly, how does social support vary according to the type of addiction? In other words, do people with substance use disorders, such as alcohol or cannabis use disorders, receive or perceive a similar level of social support to those with behavioral addictions, such as gambling and gaming? Secondly, how does social support vary in the presence of multiple addictions? The present study aimed to answer these questions by (1) assessing differences in PSS between individuals with an addiction and their peers with no addictions, (2) assessing differences in levels of PSS between types of addiction, and (3) examining how social support varied according to the number of addictions individuals had accumulated.

To answer these questions, we analyzed data from the Cohort study on Substance Use Risk Factors (C-SURF) using the same sample group and questionnaire responses examined by Tomei et al. [21]. C-SURF’s data contained valuable information for the present study’s aims. First, regarding social support, the C-SURF questionnaire included items measuring PSS rather than concrete social support [56]. Actual social support refers to the objective existence in an individual’s environment of active support from family, friends, or social workers. PSS, on the other hand, is the subjective perception of the availability and adequacy of support from intimate relationships and satisfaction with the amount and quality of that support. Significantly, it has been shown that it is specifically the perception of social support’s availability that provides its benefits, not necessarily its reality [57]. Indeed, the perception of social support’s availability seems to have a stronger impact on mental health and well-being than actually receiving it [58–60]. Notwithstanding the formal differences between actual social support and PSS, these two dimensions are, nevertheless, positively correlated [58]. Second, C-SURF’s data were collected from a large sample of male emerging adults. Emerging adulthood is defined as the period of life between 18 and 25 years old characterized by the development of greater autonomy and freedom in relation to parental monitoring and thus by more sensation-seeking and thereby risk-taking [61]. Likewise, research and prevention programs should be attentive to age and sex. It has been shown that a higher degree of PSS was associated with less substance use among male adolescents but not among their female peers [62]. A more recent study among male emerging adults went further, showing that, depending on its source, PSS may have the opposite effect on the consumption of alcohol and illicit drugs (other than cannabis). Thus, PSS from friends may amplify the risks associated with high sensation-seeking, whereas PSS from a significant other may reduce it [38]. Moreover, the association between social support and the risk of mortality was shown to be higher among males than females [5]. Third, the C-SURF questionnaire includes scales to measure addictive disorders involving multiple substances (i.e., alcohol, nicotine, and cannabis) and behaviors (i.e., gambling and gaming).

C-SURF’s large sample size and its inclusion of measurements of multiple addictions provided several advantages for answering our research questions. Indeed, we were able to compare different types of addictions (i.e., substance-related vs. behavioral addictions) and to create single-substance and single-behavior groups for evaluation. Although addiction to a single substance or behavior is not representative of most people with addiction, examining single-substance addictions might reveal specificities relating to PSS that mixtures of addictions would not. Different addiction groups were compared to a no-addiction control group. Lastly, the availability of multiple scales for measuring different types of addiction allowed us to investigate the effects of multiple addictions. Previous research has shown that addiction to multiple substances was associated with younger age [63] and greater impulsivity [64]. Regarding interpersonal relationships, other investigations assessing the personality traits of people with addiction have rated multi-substance users higher on scales for antisocial personality traits [65] and for psychosis [66] than single-substance users. This led us to hypothesize that cumulative addictions might be associated with lower-quality interpersonal relationships. To the best of our knowledge, the relationships between the number of cumulated addictions and social support had not been investigated before. We intended to address this issue via the present study. The behavioral addictions that we assessed were, therefore, included in our calculations as independent variables.

In accordance with social exchange theory and with previous reports on the relationships between social support and addiction, we tested the following hypotheses:

Hypothesis 1: We expect that PSS will be lower among respondents with an identified addiction than among controls with no addiction.

Hypothesis 2: We expect that respondents reporting problem gambling or problem gaming behaviors will show lower levels of PSS than their peers with alcohol, cannabis, or nicotine addictions. Thus, respondents with behavioral addictions like gambling and gaming will report lower levels of PSS than both controls and peers with substance-related addictions to alcohol, cannabis, or nicotine.

Hypothesis 3: We expect that PSS will decrease monotonically with the number of addictions reported by emerging adults. We have no specific hypotheses concerning the potential differences in PSS from significant others or from friends.

## Materials and methods

### Participants

Our data were collected during the second wave of C-SURF research. Participants were enrolled at three Swiss army recruitment centers when they were evaluated to determine their eligibility to serve in the military or the civilian service. As attendance at this recruitment program is mandatory for all young Swiss men around the age of 19, it provides a unique opportunity to enroll a representative sample of this population. At baseline, 7556 young men gave written informed consent to participate in the study. Although study enrolment occurred at the recruitment centers, participation in the C-SURF study took place outside of any military context [for more details on the study: 67, 68, 69]. Data collection during the study’s second wave took place between March 2012 and January 2014, when participants were 21.3 years old on average. During this period, 6020 men (79.7% response rate) completed the self-reporting questionnaire, although 366 (6.1% of respondents) were excluded due to missing values for at least one variable of interest. Thus, the final sample comprised 5654 participants (94.1% of respondents). The study was approved by Lausanne University Medical School’s Clinical Research Ethics Committee (protocol number 15/07).

### Instruments and measures

The instruments used in the present study are derived from the C-SURF survey. Given that the C-SURF included a 51-pages multi questionnaire, the selection of instruments was driven by the need to utilize short, validated scales that are widely and well-established in addiction research.

### Alcohol use disorder

Alcohol use disorder (AUD) was assessed using the eleven criteria of the Diagnostic and Statistical Manual of Mental Disorders, Fifth Edition [70]. Item questions were derived from Knight et al. [71], with an additional item specifically developed to assess craving. Our selection of the AUD tool was primarily driven by its extensive validation across diverse populations, which enhances its ability to facilitate comparisons and replication across studies. All the criteria referred to the previous 12 months. The cut-off point for determining the presence of moderate or severe AUD, as per the DSM-5, was an affirmative answer to at least four criteria.

### Nicotine dependence

Nicotine dependence (ND) was assessed using the 6-item Fagerström Test for Nicotine Dependence [FTND; 72]. The FTND is the most widely utilized scale for assessing nicotine use disorders. FTND scores range from 0–10. A score of three or more qualified as low or more severe ND [73].

### Cannabis use disorder

Cannabis use disorder (CUD) was assessed using the 10-item Cannabis Use Disorder Identification Test [74], in its validated version for Switzerland [75]. The selection of the CUDIT for this study was based on two key factors: (a) it is widely regarded as a standard and reliable brief screening tool for assessing problematic cannabis use [76], and (b) its use ensured consistency across the various waves of the longitudinal study design. The CUDIT investigates cannabis use and its consequences in the 12 preceding months. Scores range from 0–40, and, in line with Adamson and Sellman [74], the criterion for CUD was a score of 8 or more.

### Gambling disorder

Gambling disorder (GmblD) was measured based on DSM-5’s [70] 9-item diagnostic tool for detecting problem gambling in the past 12 months. Items were borrowed from DSM-IV’s Pathological Gambling Diagnostic Form (Office of Alcoholism and Substance Abuse Services, n.d.). The DSM-5 criteria is one of the main widely utilized tools for the assessment of gambling disorders that ensures consistency and reliability across studies. In accordance with DSM-5, four criteria or more answered in the affirmative indicated the presence of GmblD.

### Gaming disorder

Gaming disorder (GD) was evaluated using the shortened 7-item Game Addiction Scale [GAS; 77]. Despite its limited number of items, the GAS assesses behavioral patterns, emotional consequences, and impact of daily life. The GAS items assess seven dimensions of GD (i.e., salience, tolerance, mood modification, withdrawal, relapse, conflict, and problems). Items as about each dimension’s frequency of occurrence in the previous 6 months on a 5-point scale (1 = never, 2 = seldom, 3 = sometimes, 4 = often, 5 = very often). In line with the original scale, the criterion for GD was a reported frequency of ‘sometimes’ or more often (3 or higher) on at least 4 items.

### Perceived social support

Two dimensions of PSS—PSS from a significant other (PSS-SO) and PSS from friends (PSS-F)—were assessed using the Multidimensional Scale of Perceived Social Support [MSPSS; 78]. We have chosen the MSPSS due to its brevity, reliability and validation, as well as its long-standing widespread use. For PSS-SO, the significant other was defined as a ‘special person’, which could refer to a close, supportive friend, a romantic partner, a teacher, or a family member. Four items, evaluated on a seven-point Likert scale ranging from 1 (‘very strongly disagree’) to 7 (‘very strongly agree’), were used to measure each aspect of PSS-F (e.g., ‘My friends really try to help me’) and PSS-SO (e.g., ‘I have a special person who is a real source of comfort to me’). For PSS-F and PSS-SO, mean scores were calculated, ranging from 1–7, with higher scores reflecting higher levels of PSS.

These analyses were adjusted for age, linguistic region (i.e., French- or German-speaking), and the highest level of education achieved (i.e., obligatory schooling, vocational training, or post-secondary schooling).

## Independent variables

### Type of addiction

The type of addiction was computed as a categorical variable. Categories of the variable were defined by the following groups of participants: respondents with no measured substance or behavioral addictions (No addiction); respondents with an AUD alone (AUD); respondents with a nicotine addiction alone (ND); respondents with a cannabis addiction alone (CUD); respondents with a gambling addiction alone (GmblD); and respondents with a gaming addiction alone (GD).

### Number of addictions

The total number of addictions was calculated by adding the number of substances and behaviors that respondents were diagnosed as having. The creation of this variable led to the following categories: controls with no addictions (labeled ‘No addictions’); one addiction (coded ‘1’); two addictions (coded ‘2’); and three or more addictions (coded ‘3’).

## Statistical analyses

Descriptive statistics were calculated to characterize the sample. To compare the PSS scores of participants reporting different types of addictions with those of the no-addiction group, we created the following groups of participants: 1) AUD alone (n = 270), 2) ND alone (n = 533), 3) CUD alone (n = 187), 4) GmblD alone (n = 18), 5) GD alone (n = 360), and 6) no addictions (n = 3734). Participants reporting multiple addictions (n = 552) were excluded from these analyses. Given the aim to examine the effect of Addiction type as a unique factor on PSS while adjusting for potential confounding factors, we performed analyses of covariance (ANCOVA). Addiction type was entered as a between-subject factor, with PSS as the dependent variable and adjustments were made for age, linguistic region, and level of education. To investigate the associations between multiple addictions and PSS, a variable reflecting the number of addictions was created (i.e., none, 1, 2, and 3 + addictions). ANCOVA with the number of addictions as a between-subject factor and adjusted for age, linguistic region, and level of education were used to test differences in PSS.

### Ethics

The study was approved by Lausanne University Medical School’s Clinical Research Ethics Committee (protocol number 15/07).

## Results

Table 1 displays descriptive statistics concerning substance and behavioral addiction disorders. In our sample, 24.2% of respondents reported one addiction, and 9.8% reported two or more addictions. Nicotine addiction was the most prevalent substance-use addiction (16.7%). Among the behavioral addictions, GmblD was far less prevalent (1.1%) than GD (10.1%).

**Table 1:** Descriptive characteristics of the sample (N = 5,654).

PSS-SO (M, SD)	5.93	1.40
PSS-F (M, SD)	5.89	1.22
Prevalence of addictions (N, %)		
AUD	532	9.4
ND	943	16.7
CUD	514	9.1
GmblD	63	1.1
GD	569	10.1
Number of addictions (N, %)		
0	3,734	66
1	1,368	24.2
2	425	7.5
3+	127	2.3
Age (M, SD)	21.34	1.27
Linguistic region (N, %)		
French-speaking	3,210	56.8
German-speaking	2,444	43.2
Education (N, %)		
Obligatory schooling	433	7.7
Vocational training	2,611	46.2
Post-secondary schooling	2,610	46.2

Note. PSS-SO = Perceived social support from significant others; PSS-F = Perceived social support from friends; AUD = Alcohol use disorder; ND = Nicotine dependence; CUD = Cannabis use disorder; GmblD = Gambling disorder; GD = Gaming disorder; M = mean; SD = Standard deviation.

### PSS according to addiction type

**Fig 1:**
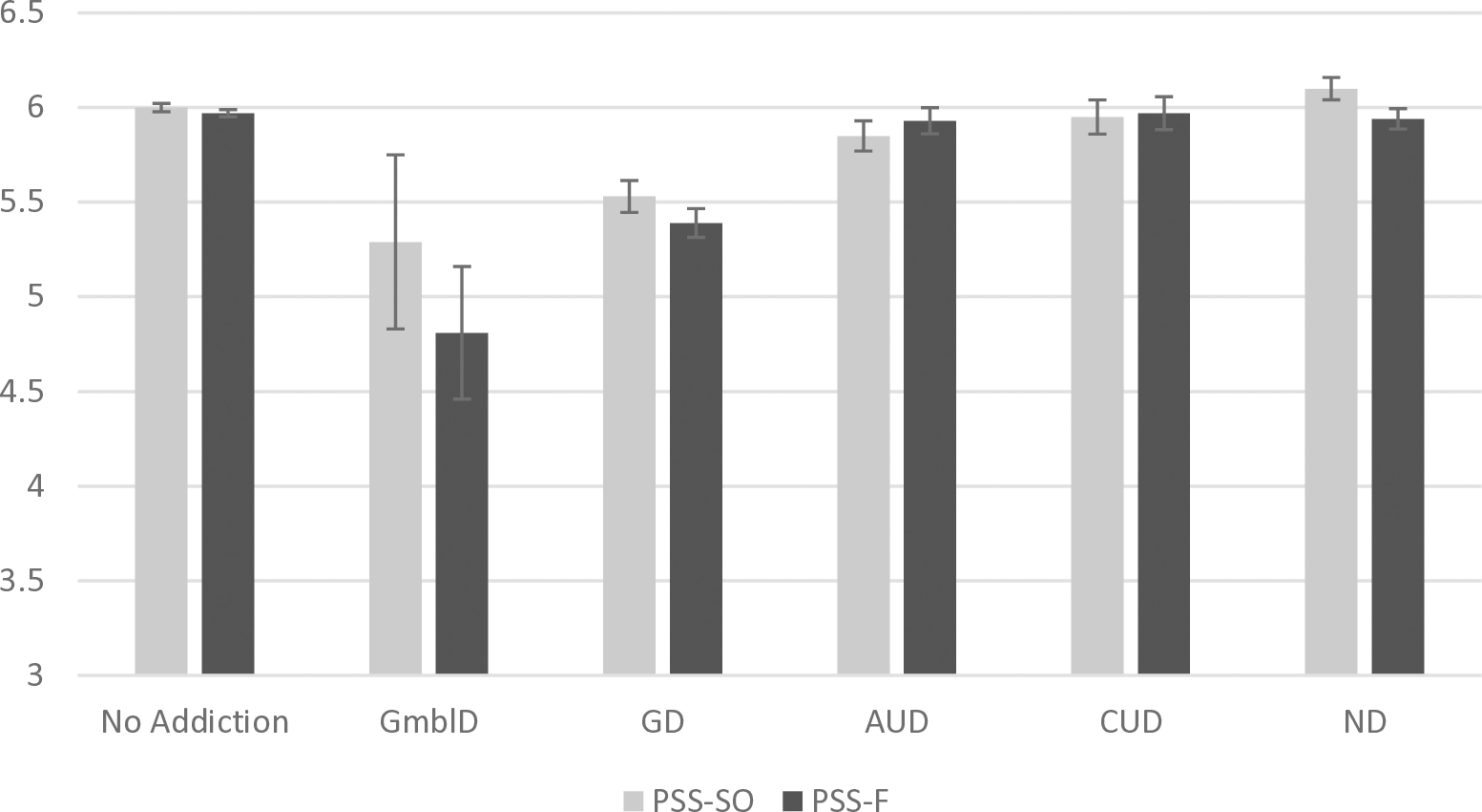
Mean PSS scores (PSS-SO = Perceived social support from significant others; PSS-F = Perceived social support from friends) according to the type of addiction. Error bars represent mean standard errors. Note. GmblD = Gambling disorder; GD = Gaming disorder; AUD = Alcohol use disorder; CUD = Cannabis use disorder; ND = Nicotine dependence.

An ANCOVA of PSS-SO showed a significant effect of the respondent’s type of addiction, *F*(5, 5092) = 9.41, *p* < .001, η_p_^2^ = .009. Fig 1 and Table 2 show pairwise comparisons indicating that individuals in the GmblD group reported the lowest PSS-SO scores, significantly lower than the scores of the no addiction, CUD, and ND groups (all *ps* ≤ .045). The GD group reported significantly lower scores than the no addiction, AUD, CUD, and ND groups (all *ps* ≤ .004). The AUD group reported significantly lower scores than the ND group (*p* = .025) but significantly higher scores than the GD group (*p* = .004). The CUD group reported higher scores than the GmblD and GD groups (all *ps* ≤ .045). The ND group reported significantly higher scores than all the other groups (all *ps* ≤ .025) except the no addiction and CUD groups.

**Table 2:** Mean PSS-SO and PSS-F scores and standard errors for each addiction group, plus a pairwise comparison matrix with between-group contrast estimates (C).

			No addiction	GmblD	GD	AUD	CUD
**PSS-SO**							
	*M*	*SE*					
No addiction	6.00	0.022	–				
GmblD	5.29	0.320	C = **.696** *p* < .030	–			
GD	5.54	0.072	C = **.457** *p* < .001	C = .238 *p* = .467	–		
AUD	5.85	0.083	C = .145 *p* = .091	C = .551 *p* = .096	C = **.312** *p* = .004	–	
CUD	5.97	0.099	C = .024 *p* = .810	C = **.671** *p* = .045	C = **.433** *p* < .001	C = .120 *p* = .353	–
ND	6.09	0.059	C = .084 *p* = .186	C = **.780** *p* < .017	C = **.541** *p* < .001	C = **.229** *p* = .025	C = .109 *p* = .348
**PSS-F**							
	*M*	*SE*					
No addiction	5.96	0.019	–				
GmblD	4.83	0.277	C = **1.114** *p* < .001	–			
GD	5.41	0.062	C = **.544** *p* < .001	C = **.570** *p* = .045	–		
AUD	5.92	0.072	C = .046 *p* = .533	C = **1.068** *p* < .001	C **=** **.498** *p* < .001	–	
CUD	6.00	0.086	C = .049 *p* = .580	C = **1.163** *p* < .001	C = **.593** p < .001	C = .095 *p* = .397	–
ND	5.95	0.051	C = .011 *p* = .842	C = **1.103** *p* < .001	C = **.533** *p* < .001	C = .0.35 *p* = .691	C = .060 *p* = .551

Note. Significant contrast estimates are noted in bold text. GmblD = Gambling disorder; GD = Gaming disorder; AUD = Alcohol use disorder; CUD = Cannabis use disorder; ND = Nicotine dependence; C = Contrast estimates between addiction type by line and addiction type by column.

An ANCOVA of PSS-F also showed the significant effects of the respondent’s type of addiction, *F*(5, 5092) = 17.18, *p* < .001, η_p_^2^ = .017. Fig 1 and Table 2 show pairwise comparisons indicating that individuals in the GmblD group reported the lowest PSS-F scores, significantly lower than the scores of all the other addiction groups, including the no-addiction group (all *ps* ≤ .045). The GD group reported significantly lower scores than the no-addiction, AUD, CUD, and ND groups (all *p* *< *.001) but higher scores than the GmblD group (*p* = .045). AUD, CUD, and ND groups reported significantly higher scores than the GmblD and GD groups (*p* < .001).

### PSS according to the number of addictions

**Fig 2:**
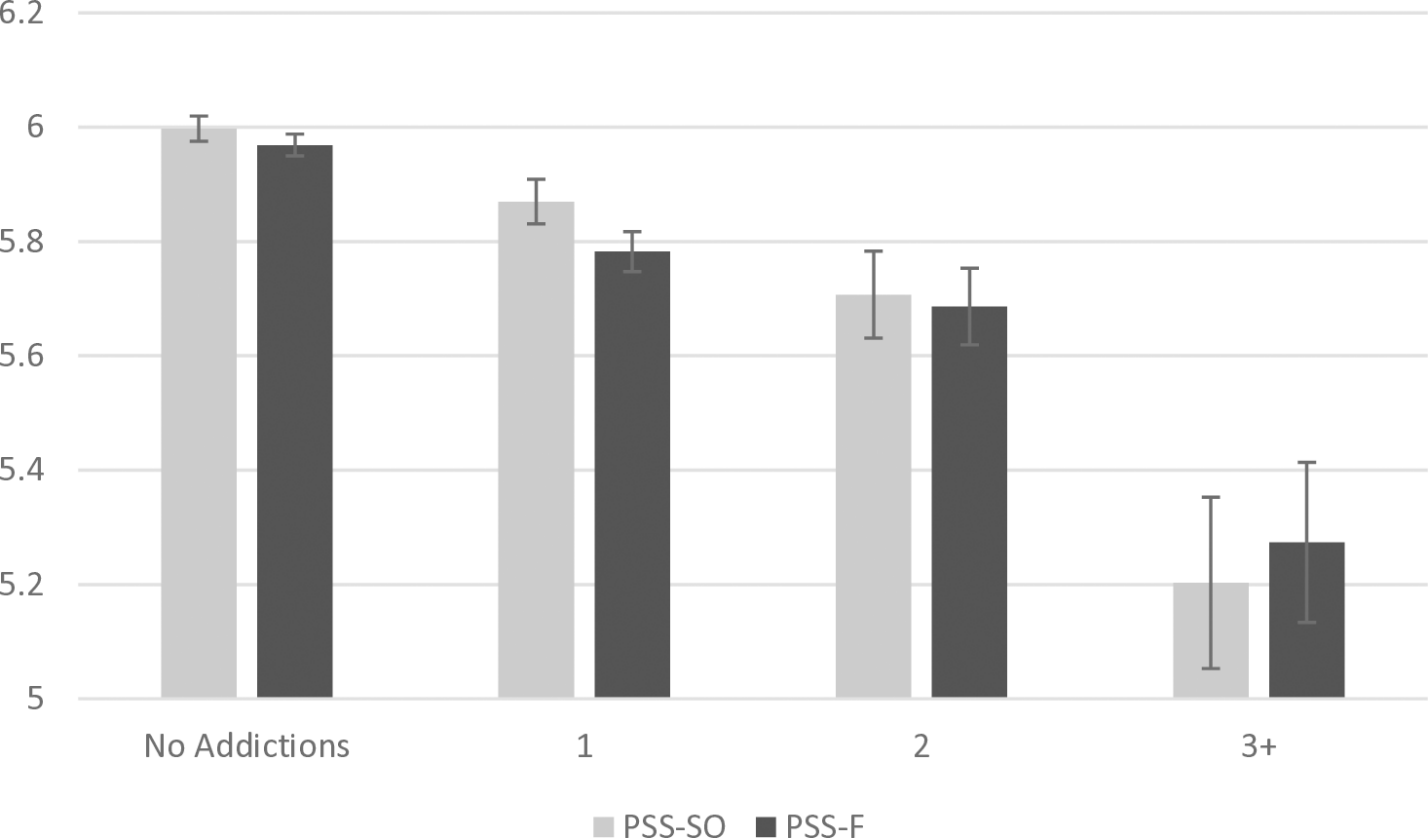
Mean PSS-SO and PSS-F scores according to the number of addictions. Error bars represent mean standard errors.

Our ANCOVA showed that differences in the number of addictions significantly affected PSS-SO scores (*F*(3, 5646) = 17.92, *p* < .001, η_p_^2^ = .009), and with significant linearity (*p* < .001). As Fig 2 and Table 3 show, pairwise comparisons indicated that individuals reporting one or more addictions had significantly lower PSS-SO scores than those reporting no addictions (all *ps* ≤ .005). Individuals reporting two and three or more addictions had significantly lower PSS-SO scores than those reporting one addiction (all *ps* ≤ .026), and those individuals reporting three or more addictions had significantly lower scores than those reporting two addictions (*p* < .001).

**Table 3:** Mean group PSS-SO and PSS-F scores and standard errors based on numbers of addictions, and a pairwise comparison matrix with between-group contrast estimates (C).

			No addiction	1	2
**PSS-SO**					
	*M*	*SE*			
No addiction	6.00	0.023	–		
1	5.87	0.038	C = **.124** *p* = .005	–	
2	5.71	0.068	C = **.295** *p* < .001	C = **.171** *p* = .026	–
3+	5.20	0.123	C = **.770** *p* < .001	C = **.646** *p* < .001	C = **.475** *p* = .001
**PSS-F**					
	*M*	*SE*			
No addiction	5.96	0.020	–		
1	5.78	0.033	C = **.159** *p* < .001	–	
2	5..69	0.059	C = **.249** *p* < .001	C = .090 *p* = .178	–
3+	5..27	0.107	C = **.628** *p* < .001	C = **.469** *p* < .001	C **= .379** *p* = .002

Note. Significant contrast estimates are noted in bold text. C = Contrast estimates between addiction type by line and addiction type by column.

Similarly, our ANCOVA showed that differences in the number of addictions significantly affected PSS-F scores (*F*(3, 5646) = 18.29, *p* < .001, η_p_^2^ = .010), and with significant linearity (*p* < .001). As Fig 2 and Table 3 show, pairwise comparisons indicated that individuals reporting no addictions had significantly higher PSS-F scores than those reporting one or more addictions (all *p*s ≤ .001). Individuals reporting three or more addictions had significantly lower PSS-F scores than those reporting one addiction (*p* < .001), and individuals reporting three or more addictions had significantly lower scores than those reporting two addictions (*p* = .002).

## Discussion

The present study’s results showed relatively high prevalence rates of the addictions considered. Indeed, high rates of addictive disorders are quite representative of young male populations in general. In Switzerland, it has already been shown that demographic characteristics such as being a young man and residing in the country’s French-speaking regions are associated with the problematic use of alcohol [79], cannabis [80], gambling [81,82], and gaming [83].

Our first hypothesis—that reported PSS scores would be smaller among male emerging adults with an addiction than their peers with no addictions—was confirmed in part. Indeed, significant differences from the control group’s PSS-SO and PSS-F scores were only observed for the GmblD and GD groups. These findings confirmed previous reports showing an association between GmblD [49–52] and GD [53]. The scores of respondents reporting AUD, CUD, or ND alone did not differ from those of their control group peers. Regarding AUD and CUD, the present findings contradict those of previous reports showing less social support for individuals with AUD [37] or CUD [84] than for individuals with no addictions. On the contrary, regarding ND, our findings were in line with those of previous reports attesting to a relationship between smoking and social support, with smoking enhancing social functioning rather than disrupting it [85].

Our second hypothesis—that participants with behavioral addictions would report lower levels of PSS than their peers with substance addictions—was confirmed. Except for the participants with GmblD not differing significantly from those with AUD (probably due to low prevalence and wide confidence intervals for PSS-SO scores), the GmblD and GD groups reported lower levels of PSS than the other substance disorder groups, both for PSS-SO and PSS-F scores [84].

Our third hypothesis—that PSS scores would decrease monotonically with the number of addictions reported—was also confirmed. As the results clearly showed, PSS-SO and PSS-F scores decreased monotonically from the no-addiction group to the one-addiction group and to the two-addiction group. However, PPS-SO and PSS-F scores decreased still further among participants reporting three or more addictions. This finding suggested that because increasing numbers of concurrent addictions corresponded to a greater overall pervasiveness of addiction and a lower potential for recovery overall [86], significant others and friends seemed to become less involved in supporting their loved ones struggling with two, three, or more addictions. Furthermore, significant others and friends did not seem to differ in this respect. The trend of a monotonic decrease in PSS score as the number of addictions increased was similar to its association with the level of prosocialness among the same population [21]. This supported the idea that, as previously suggested, an extended imbalance in interpersonal exchanges may threaten even the closest of relationships [87]. Finally, the pattern of the associations with PSS-SO and PSS-F were similar since no significant differences were found between these two measurements.

Interestingly, the present study’s results seem to concur with those of Tomei et al. [21]. Indeed, Tomei et al. [21] showed that male emerging adults with behavioral disorders like GmblD and GD reported lower levels of prosocial behaviors than controls with no addictions and their peers with AUD, CUD, or ND. Tomei et al. [21] also showed that participants with GmblD reported lower levels of prosocialness than those with the other types of addictions considered. The parallels between the results of these two studies suggest that individuals with GmblD and GD have fewer relationships involving reciprocal exchanges with their entourage than do individuals with substance-related addictions such as AUD, CUD, or ND. Evidence from previous reports has shown that individuals with behavioral addictive disorders have poorer interpersonal relationships [88], less empathy [17,19], and fewer prosocial behaviors [21]. This suggests that even friends and family may find it demanding and distressing to maintain support for their friend or relative with a gambling or gaming condition when they expect no reciprocal behavior in return. Healthy, well-balanced relationships clearly cannot persist with this kind of rapport, and they may even be put in peril. It is not a coincidence that the DSM-5’s diagnostic criteria for disordered gambling (but not for the other addictive disorders) include a socially-related criterion according to which the person with problematic gambling behaviors “may have jeopardized or lost a significant relationship” [70]. Indeed, socially well-adapted interpersonal relationships imply that peers exchange benefits based on the concept of equity [89,90]. People do not generally choose to invest in relationships that are more costly than they are rewarding [91].

In short, the present findings suggested that, particularly among male emerging adults with gambling or gaming problems—and to a lesser extent among those with a problematic AUD—PSS from family and friends should be given particular attention in diagnostic and treatment programs. Indeed, lower-quality social relationships are a major determinant of physical and mental health. Social support also has an impact on health behaviors [35], health-promotion behaviors [92,93], and treatment retention [94–97]. Addiction treatment programs, therefore, should include the promotion of social connections in order to reduce deteriorations in health and the risk of premature mortality. Our findings also suggested that the association between PSS and having multiple addictions requires more attention. Indeed, although distinguishing between multiple addictions and single addictions may play an important role in treatment policies [63], considering the association between multiple addictions and PSS may also contribute to providing more informative diagnoses.

The present study nevertheless had several limitations. One was the small size of the gambling addiction group, which requires caution in interpretation. The limited sample size may lead to increased instability in the parameters, reducing precision and increasing variability in the estimates, thereby affecting the statistical power and reliability of our findings. Another limitation was that the types of addictions being compared were limited, as other addictions were not assessed (e.g., cocaine use disorder, heroin use disorder). Further, the sample only included young men. This raises the question of whether the trends in PSS that emerged in the study are limited to young men or could be observed among young women and across other age categories. Also, the sample consisted solely of Swiss nationals, which introduces a bias regarding cultural diversity of the population with potential implications on the addiction – perceived social support relationship. An additional limitation is that the cross-sectional design of the study limits the ability to establish causal relationships between the examined variables. As such, the present study cannot determine whether the perceived social support is a cause of addiction or merely a consequence of it. For instance, low social support may contribute to addiction by fostering isolation and loneliness, while addiction may, in turn, lead to reduced support due to social withdrawal or relationship strain. This underscores the need for longitudinal studies to better understand the directionality of this relationship. Finally, another limitation is that the study did not account for potential comorbidities that may be associated with the severity of addiction in the sample.

Future research could thus extend the examination of relationships between PSS and addictions to other population categories, including women and other age and cultural groups. That research could also extend to other substance and behavioral addictions. Another avenue of research worth pursuing might be to look into whether other societal factors, including loneliness, might confirm the socially related specificities of gambling and gaming disorders compared to other types of addiction. Indeed, loneliness has been shown to be associated with PSS among people with addictive disorders [29] and to be an important explanatory factor of illness and mortality [98].

The present study provided new evidence on the social dimensions affecting male emerging adults with addictive disorders. It showed that individuals with behavioral disorders such as addictions to gambling and gaming reported lower PSS from significant others and friends than did young men with alcohol, cannabis, or nicotine disorders. One potential explanation is that behavioral addictions are more strongly associated with social anxiety than substance-related addictions [99]. Additionally, recent studies have demonstrated that social anxiety is linked to both higher levels of mobile phone addiction and lower perceived social support [100].

The study also showed that PSS decreased as the number of addictions increased. As these findings exhibited the same trends as those found by Tomei et al. [21] regarding prosocialness, they suggested that the social exchange dynamics of male emerging adults with gambling and gaming addictions are particularly affected. In terms of social exchange theory, the diminished frequency of social behaviors and reduced social support imply that these individuals, more than those with substance-related addictions, might experience a withdrawal of social investment from their environment. This, in turn, may put them at risk of isolation and solitude and thus of incurring a deterioration in their physical and mental health. As these addictive disorders are already subject to significant social stigma [101–103] and social distancing [104], it is important that the social and relational dimensions of addiction be included in treatment and rehabilitation programs in order to maintain or enhance individuals’ sense of social belonging and social integration [105] and thus their physical and mental health. Treatment approaches may involve exploring the emotional and relational foundations of addiction and modifying dysfunctional thought patterns. Interventions may focus on enhancing social skills, strengthening social support, and increasing opportunities for social interaction. Specifically, interventions targeting dysfunctional social cognitions have shown to be particularly effective [106]. Regardless of the therapeutic approach or specific intervention employed, it is essential to incorporate social factors into the treatment plan to support recovery and promote long-term well-being.
